# Gujarat's *Chiranjeevi Yojana* – a difficult assessment in retrospect

**DOI:** 10.2471/BLT.14.137745

**Published:** 2015-05-05

**Authors:** Ayesha de Costa, Kranti Vora, Eric Schneider, Dileep Mavalankar

**Affiliations:** a Public Health Sciences, Karolinska Institutet, Tömtebodavägen 18A, 17177 Stockholm, Sweden.; b Indian Institute of Public Health, Ahmedabad, India.; c Johns Hopkins Bloomberg School of Public Health, Baltimore, United States of America.

The *Chiranjeevi Yojana* programme in Gujarat, India, promotes institutional childbirth among poor and tribal women. The retrospective evaluation of the programme, by Mohanan et al., has some limitations.^1^

The five early implementing districts are socioeconomically and demographically different from the 21 later implementing districts,^2^ leading to unmeasured confounding. Also, the wide confidence intervals in table 2 are of concern, as they suggest that the sample of births in each district may have been too small to adequately assess variability at the district level. The authors do not report the overall or annual number of births per district. If we assume that there was one birth per surveyed household and that these births were evenly distributed over each of the five years, we would estimate an average of 250 births over five years or 50 births per year, per district. This may explain the wide confidence intervals and large standard deviations reported. An average sample of 50 births per year in each district may be inadequate to capture the variability both within and between districts and could potentially lead to biased conclusions.

As the sample consisted of households with a poverty score ranging from 16 to 25, the sampling method excluded the poorest households (those scores less than 16), the group that would be most likely to benefit from the *Chiranjeevi Yojana* programme. In 2012, about 17% of the population lived below the poverty line in Gujarat.^3^ Also, a higher proportion of impoverished households exist in the early implementing districts than in the 21 comparison districts. Systematically excluding these households would also introduce a bias.

The use of self-reports for obstetric complications may also lead to bias.^4^^,^^5^ The study design is based on differences occurring over short time periods: in the year between the start of the programme in the early and late implementing districts. However, *Chiranjeevi Yojana* was still establishing itself in those districts. Data on deliveries under the *Chiranjeevi Yojana* programme in Gujarat indicate that these climbed steadily for the first two to three years following implementation.^6^ As the authors write, overall institutional delivery has increased in Gujarat since the *Chiranjeevi Yojana* programme was implemented. Government statistics indicate a rise from 50% to 90% between 2005 and 2010.^7^^,^^8^ Much of this rise is attributable to an increase in private sector deliveries. We are concerned that the large secular increase in the proportion of institutional deliveries might obscure an even larger proportional increase in institutional deliveries among the poor. This is because the programme specifically targets households below the state-specific poverty-line (of 15) which represented 23% of the population in Gujarat in 2010.^9^ The increase among the poor might be difficult to detect in the small samples examined in this study. It is also unclear why the authors chose the entire District Level Household Survey-3 sample of women who gave birth after 2005 and not just the population targeted by the programme. Perhaps this was due to difficulties in obtaining a sufficient sample size of women in this stratum, but this broad inclusion could substantially bias results.

For these reasons, we suspect that the authors’ samples and methods may have led to a type II error – i.e. the real effect was disregarded.

Many programmes are scaled up by governments before evidence is generated of their effectiveness. If evaluation is omitted before scaling up, a relevant well-functioning health management information system will allow some estimation of the programme’s impact. The impact can be estimated by comparing selected indicators in the target group before and after the programme is implemented. For the *Chiranjeevi Yojana* programme, obtaining relevant indicators from the health information system would have been problematic because maternal deaths are rare events that tend to be underreported

Our recent assessment^6^ based on secondary data obtained from the Department of Health and Family Welfare, Gujarat, India, indicates that approximately a third of all institutional deliveries in the target group occurred under the *Chiranjeevi Yojana* programme. However, the variations between districts were large. Despite covering nearly a million deliveries, *Chiranjeevi Yojana* could still increase its coverage of eligible beneficiaries. It would be useful to investigate where the greater proportion of eligible women deliver and what barriers they face to entering the programme. Our assessment indicates that between 2006 and 2010, 6% of deliveries provided by *Chiranjeevi Yojana* were caesareans.^6^ This is higher than before the implementation of the programme, when 2% of women in the two lowest wealth quintiles reported a caesarean delivery in the DLHS survey.^10^ We cannot assess if deliveries have shifted from home, government facilities or paying private facilities to the *Chiranjeevi Yojana* programme, as these data were not recorded. Nevertheless, from the women's perspective, delivery under the programme is likely to represent a shift to safe delivery facilities where functional emergency obstetric care is available at lower cost.
